# Mathematical Model of Muscle Wasting in Cancer Cachexia

**DOI:** 10.3390/jcm9072029

**Published:** 2020-06-28

**Authors:** Suzan Farhang-Sardroodi, Kathleen P. Wilkie

**Affiliations:** Department of Mathematics, Ryerson University, 350 Victoria St, Toronto, ON M5B 2K3, Canada; suzan.farhang@ryerson.ca

**Keywords:** cancer cachexia, muscle wasting, mathematical oncology, mathematical modeling, dynamical systems

## Abstract

Cancer cachexia is a debilitating condition characterized by an extreme loss of skeletal muscle mass, which negatively impacts patients’ quality of life, reduces their ability to sustain anti-cancer therapies, and increases the risk of mortality. Recent discoveries have identified the myostatin/activin A/ActRIIB pathway as critical to muscle wasting by inducing satellite cell quiescence and increasing muscle-specific ubiquitin ligases responsible for atrophy. Remarkably, pharmacological blockade of the ActRIIB pathway has been shown to reverse muscle wasting and prolong the survival time of tumor-bearing animals. To explore the implications of this signaling pathway and potential therapeutic targets in cachexia, we construct a novel mathematical model of muscle tissue subjected to tumor-derived cachectic factors. The model formulation tracks the intercellular interactions between cancer cell, satellite cell, and muscle cell populations. The model is parameterized by fitting to colon-26 mouse model data, and the analysis provides insight into tissue growth in healthy, cancerous, and post-cachexia treatment conditions. Model predictions suggest that cachexia fundamentally alters muscle tissue health, as measured by the stem cell ratio, and this is only partially recovered by anti-cachexia treatment. Our mathematical findings suggest that after blocking the myostatin/activin A pathway, partial recovery of cancer-induced muscle loss requires the activation and proliferation of the satellite cell compartment with a functional differentiation program.

## 1. Introduction

Cancer cachexia is a condition identified by an ongoing and irreversible loss of skeletal muscle and adipose tissue [1,2,3,4]. Depending on the tumor type, cachexia affects 30 to 85% of cancer patients [5], and it leads to increased morbidity and mortality [6,7,8]. The highest incidence of cachexia is seen in patients with pancreatic, gastric, or lung cancers [9,10]. In pancreatic cancer, over 85% of patients suffer from cachexia, contributing to its very poor survival statistics [11]. Historically, the cause of cancer cachexia was thought to be metabolic dysregulation [9,12], which led to nutritional- and fitness-based therapeutic approaches. None of these approaches were successful, however, leaving cachexia as an untreatable condition that severely impacts patient quality of life [13,14,15]. Extreme weight loss is the primary clinical feature by which cachexia is defined. Generally, an unintended body weight loss of more than 5% in a year is considered cachexia [1,5]. The dramatic weight loss causes increased disease symptoms such as pain, weakness, and fatigue [16] and leads to limited treatment options [17,18].

Cancer cachexia is now known to be strongly associated with systemic inflammation [19]. Pro-inflammatory cytokines including tumor necrosis factor α (TNFα) and interleukins 1, 6, and 10 (IL-1, IL-6 and IL-10) have been associated with the cachectic phenotype in several cancer models [20,21,22,23]. These cytokines activate the STAT3 and NF-κB signaling pathways, leading to protein degradation [11,24,25]. Immune infiltrates such as tumor-associated macrophages are believed to be key reservoirs of these cytokines. Indeed, conditioned media from macrophages and pancreatic cancer cells have been shown to promote muscle atrophy synergistically through STAT3 signaling [24]. Furthermore, it was observed that macrophage depletion attenuated systemic inflammation and muscle wasting in pancreatic tumor-bearing mice. Another study showed that brain inflammation in a mouse model of pancreatic cancer may drive pancreatic ductal adenocarcinoma-associated anorexia, which contributes to the cachectic phenotype [26].

Recently, a new paradigm in cancer cachexia treatment has arisen, proposing that tumor-derived factors may alter tissue behavior through molecular signaling. Various tumor-derived factors such as the cytokines mentioned above, hormones, and glucocorticoids can influence muscle protein balance through signaling transduction pathways [27]. Several treatments have been proposed to target upstream mediators of inflammation or downstream factors in catabolic/anabolic muscle pathways [28]. In particular, the myostatin/activin A signaling pathway plays a significant role in cancer-induced muscle atrophy [29,30,31,32,33]. Indeed, blocking the activin type-2 receptor (ActRIIB) with a soluble decoy receptor (sActRIIB) or a myostatin antibody in several mouse models of cachexia has been shown to prevent muscle wasting and also to reverse the prior loss of skeletal muscle [31,34,35,36,37]. Consequently, the survival time of the tumor-bearing animals was increased, even though the tumor itself was not therapeutically targeted.

Myostatin and activin A are members of the transforming growth factor-β (TGF-β) superfamily of proteins. They both bind to ActRIIB, a high affinity receptor [38,39,40,41], resulting in the phosphorylation of SMAD2 and dephosphorylation of FOXO3a, which consequently induces satellite cell quiescence. The dephosphorylated FOXO3a then moves into the nucleus and induces two muscle-specific ubiquitination ligases that facilitate the degradation of myofibrillar proteins, leading to muscle loss and cachexia [10,31,42,43,44,45].

Recent work by Zhou et al. [31] demonstrated that blocking the activation of ActRIIB and the subsequent downstream signaling events could stop muscle loss in cancerous mice. They assessed the effects of an ActRIIB decoy receptor in several mouse models of cancer cachexia, including a murine colon-26 carcinoma and two xenograft models: human G361 melanoma and human TOV-21G ovarian carcinoma. The decoy receptor sActRIIB potentially prevents binding of myostatin and activin A to the ActRIIB receptor and inhibits the downstream signaling. In each case, treatment with sActRIIB was found to prevent or reverse skeletal muscle atrophy and heart muscle wasting, although it had no effect on adipose tissue loss or the production of proinflammatory cytokines (for C26 tumor bearing mice).

Under normal conditions, adult skeletal muscle preserves its mass via feedback mechanisms that balance the signals controlling muscle catabolism and anabolism. When signaling is dysregulated by tumor-derived factors, the implications on tissue homeostasis are not clear due to the nonlinear and complex dynamics involved in such feedback control mechanisms. Therefore, a quantitative tool is needed to better understand the significant factors regulating muscle atrophy (increased muscle protein degradation) and hypertrophy (increased muscle protein synthesis). Mathematical modeling is one such tool, and the predictive nature of the model presented in this paper can be used to examine the complex nature and potential treatments of cancer cachexia.

Mathematical oncology is a burgeoning field of research aimed at constructing predictive models that provide new insights into the underlying mechanism of cancer initiation, progression, metastasis, and treatment. Due to the complexity of this disease, it has been suggested that conceptual frameworks are needed to interrogate the data provided by laboratory experiments and clinical trials [46,47,48]. Cancer modeling involves a wide range of mathematical formalisms, including ordinary differential equations, partial differential equations, cellular automata, and hybrid approaches, which are used to examine all aspects of the disease evolution and treatment [49,50,51,52,53,54]. Mathematical models of muscle degradation and regeneration have been proposed to study muscular dystrophies [55]. In [56], a new mathematical model was presented to explore the healing process of healthy skeletal muscle. In cancer cachexia, the modeling work is limited and focuses on metabolic alterations and energy expenditures [57,58]. Recently, a model of tumor-promoting inflammation was proposed [59], which may be a key driver of cancer cachexia. To the best of our knowledge, we are the first to present a mathematical model of muscle degeneration in the context of cancer cachexia.

We present a novel mathematical framework to explore the effects of dysregulated signaling in cancer cachexia. Inspired by a model for population growth with negative feedback [60], we construct a model of feedback-regulated healthy muscle tissue by describing the growth rates of muscle and satellite cells (muscle stem cell) compartments using ordinary differential equations. Negative feedback from the muscle to stem compartments maintains tissue homeostasis. We then extend the model to include a growing tumor that interferes with the intercellular signals and, thus, with the feedback control. This dysregulation leads to muscle loss in the model. Finally, the model is extended to simulate the effects of ActRIIB blockade treatment. Model parameters are found by fitting to experimental data for healthy, diseased, and therapeutic states, and the model is validated by comparing to a second experimental group [31]. Model simulations suggest that satellite cell reactivation is the primary target for muscle recovery through the myostatin/activin A/ActRIIB signaling pathway.

## 2. A Model of Healthy Muscle Tissue

Skeletal muscle is a form of striated muscle tissue, accounting for about 40% of a healthy person’s body weight [61]. Adult skeletal muscle, which is stable under normal circumstances, has an extraordinary ability to regenerate post-injury by activating various precursor cells. Here, we focus on the dynamics of satellite cells (the principal skeletal muscle stem cells [62]) and myofibers (the terminal differentiated muscle cells). Satellite cells comprise approximately 3–7% [63,64,65,66] of all muscle nuclei in skeletal muscle and are mitotically dormant unless activated by an external signal. Upon receiving an activation signal, a satellite cell will enter the cell cycle to proliferate and differentiate into myogenic precursor cells in an attempt to repair the damage and maintain tissue homeostasis [67,68,69,70,71,72].

To model the dynamics of skeletal muscle regeneration and repair, we begin with a classic model of cell lineage [60,73]. Neglecting the intermediate precursor cells to avoid unnecessary complexity, we consider only two cell compartments: the satellite cells (or stem cells) S(t) and the muscle cells (or differentiated cells) M(t):

(1) $$\left\{\begin{array}{c} \frac{dS}{dt}=\left(2p\left(t\right)-1\right)\nu \left(t\right)S\left(t\right),\\ \frac{dM}{dt}=2\left(1-p\left(t\right)\right)\nu \left(t\right)S\left(t\right)-d_{0}M\left(t\right). \end{array}\right.$$

Here, $d_{0}$ is the natural death rate for differentiated muscle cells. The proliferation rate of satellite cells is ν(t), and the probability of symmetric self-renewing division is p(t); that is, once activated, a satellite cell will divide into two daughter satellite cells with probability p(t) and into two differentiated muscle cells with probability 1−p(t). The probability of self-renewal, p(t), and the proliferation rate, ν(t), are determined by intercellular interactions and thus provide the mechanism for negative feedback control. The muscle compartment will decrease the proliferation rate and probability of self-renewing division as it approaches its homeostatic level. Following muscle injury, satellite cells undergo extensive proliferation to self-renew and differentiate into muscle cells [74]. After resolution of the healing process, the division rate and probability return to their homeostatic maintenance levels. To model feedback in the symmetric division probability, we propose:

(2) $$p\left(t\right)=p_{0}+\frac{p_{1}}{1+\frac{1}{m}M\left(t\right)}$$

where $p_{0}$ is the homeostatic probability, $p_{1}$ is the perturbation probability activated in growth or in response to injury, and *m* is the half-saturation constant. Since this is a probability, we must have $0<p_{0}+p_{1}\leq 1$. Notice from (1) that if ν(t)>0, then $p\left(t\right)>\frac{1}{2}$ will result in unbounded growth in the stem compartment, whereas $p\left(t\right)=\frac{1}{2}$ will result in no growth. We thus expect $p\left(t\right)\approx \frac{1}{2}$ in the steady-state.

Similar to the above, we propose the same feedback form for the proliferation rate of satellite cells:

(3) $$\nu \left(t\right)=\nu_{0}+\frac{\nu_{1}}{1+\frac{1}{m}M\left(t\right)}.$$

Here, $\nu_{0}$ is the homeostatic division rate, $\nu_{1}$ is the perturbation division rate activated in growth or in response to injury, and *m* is the half-saturation constant.

These negative feedback loops provide two mechanisms by which the satellite cell behavior is controlled by the muscle compartment. Together, they act to regulate the tissue to maintain homeostasis.

### 2.1. Model Parameterization

The experimental data that were available to us came in many forms: body or tissue mass in grams (g), tumor volume in mm${}^{3}$, and cell numbers (from tumor implantation). We thus needed to manipulate the data to match our mathematical model description, which was nominally a volume measurement for each compartment. To parameterize our model, we deconstructed body weight measurements into an estimate of lean muscle mass. We assumed that body weight (mass) consisted of 40% skeletal lean mass and 60% other (including adipose tissue, bone, organ tissue, etc). From this 40%, we estimated that 95% was due to muscle cells and 5% due to satellite cells. Finally, to convert mass in grams to tissue volume in mm${}^{3}$, we estimated a conversion factor of ξ=0.002 g/mm${}^{3}$ (from an estimated tumor volume of 1000 mm${}^{3}$ weighing 2 g [31] (Figure 2C)). These breakdowns were estimates that allowed us to fit our model to the experimental data. All numerical simulations were conducted with default numeric integration methods using either NDSolve in Mathematica (www.wolfram.com/mathematica/) or Dsolve in Maple (www.maplesoft.com), which are similar numerical solvers for initial value problems. A sample Mathematica script to plot the numeric solution of the model is provided in Appendix A.

### 2.2. Healthy Experimental Data

**Figure 1:**
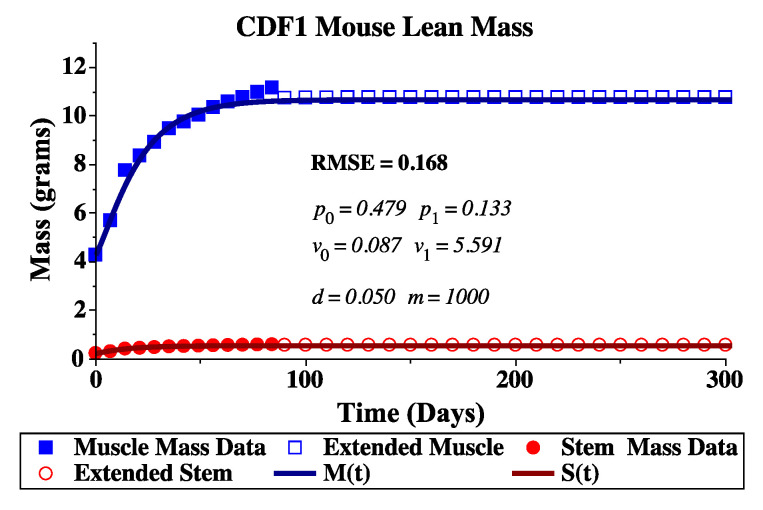
Lean body mass for a U.S.-bred CDF1 male mouse. Body weight measurements from the suppliers’ published growth data [75] are deconstructed into estimated lean mass (40% of body weight) and then further divided into muscle (95%, solid box) and stem (5%, solid circle) lean masses. The lean mass data is extended by fitting a logistic growth model to average body weight which is then deconstructed into estimated lean mass (40% of body weight) and then divided into muscle (95%, open box) and stem (5%, open circle) lean masses. Finally, these extended muscle and stem mass datasets are used to fit the mathematical model given by Equations (1)–(3) in Maple.

To parameterize the healthy feedback control mechanisms that exist in normal growth and development, we fit our model (1)–(3) to average body weight measurements of the U.S.-bred male CDF1 mouse. Growth data were available from the supplier for the first 3 to 15 weeks of life [75], which did not fully characterize the adult mouse size. We thus extended these data to 300 days by fitting a logistic growth curve to the average body weight W(t):

(4) $$\frac{dW}{dt}=\alpha W\left(t\right)\left(1-\frac{W(t)}{K}\right)$$

with a growth rate of α=0.0756, a carrying capacity of K=28.3 g, and an initial mass at 3 weeks, Day t=0, of W(0)=11.26 g. The fit of this logistic curve to the body weight data was computed in Mathematica using the NonlinearModelFit command. Actual experimental data points were then extended beyond 15 weeks (84 days), by sampling from this logistic growth model every 10 days from Day 90 up to Day 300. Fitting to the extended dataset ensured the model parameters captured the homeostatic adult muscle mass size, which was not fully realized in the actual dataset. Experimental and extended body weight data converted to lean body mass (40% of body weight divided into 95% muscle cells and 5% stem cells) are shown in Figure 1.

#### Simulated Annealing for Parameter Fitting

The full feedback model defined by Equations (1)–(3) is difficult to parameterize due to the nonlinear structure and high sensitivity around $p\left(t\right)=\frac{1}{2}$. As discussed above, if $p\left(t\right)>\frac{1}{2}$ then unbounded growth occurs in the stem compartment, which is not physical. In the steady-state, we expected the stem compartment to be approximately constant, and this requires $p\left(t\right)\approx \frac{1}{2}$. The restrictions on $p_{0}$ make it highly sensitive to change. This sensitivity arose from the decision to consider only symmetric self-replication or symmetric differentiation of stem cells in the model. Potentially, the model could be extended to allow satellite cell asymmetric division with a probability that increases to 1 near the steady-state to remove this high sensitivity. The addition of a third division probability of satellite cells would increase the complexity of our model and is left for future exploration.

Parameterization was thus performed hierarchically by fixing two model parameters (*d* and *m*) and using a simulated annealing algorithm [76,77] to fit the remaining parameters ($p_{0}$, $p_{1}$, $\nu_{0}$, and $\nu_{1}$) that control the feedback mechanisms. After a successful fit, parameters *d* and *m* were varied one at a time, and simulated annealing was used to find the remaining parameters again. The final fit was determined by the minimum of our objective function, the root-mean-squared-error (RMSE), which considered differences from both estimated and extended muscle and satellite mass data and the model predicted values. The parameter values determined in this manner are listed in Table 1. The model fit to the data is shown in Figure 1.

## 3. Modeling Cancer Cachexia and Treatment

**Figure 2:**
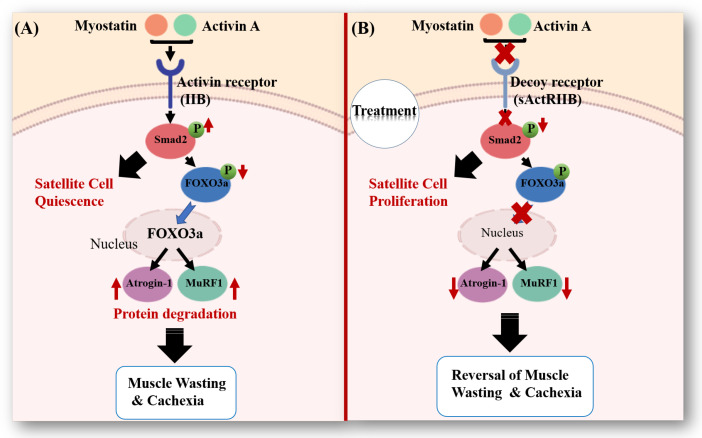
Myostatin and activin signaling pathway in muscle. (**A**) Myostatin and activin A bind to the activin type IIB receptor (ActRIIB) on muscle cell membranes, resulting in phosphorylation of Smad2. The phosphorylated Smad2 induces satellite cell quiescence and the dephosphorylation of transcription factor FOXO3a, which consequently moves into the nucleus and activates the transcription of E3 ubiquitin ligase, MuRF1, and Atrogin-1. These muscle-atrophy enzymes provide the specificity that causes the degradation of muscles leading to cachexia. (**B**) Using a soluble form of the activin type IIB receptor, sActRIIB, the effect of myostatin/activin A signaling is blocked, resulting in a decrease in phosphorylated Smad2, an increase in satellite cell proliferation, and a decrease in ubiquitin enzymes. Blocking this pathway can lead to muscle hypertrophy and reversal of cachexia [45].

Myostatin and activin A are two promising molecular targets of muscle wasting in cancer cachexia [27,78]. Myostatin and activin, from the TGF-β superfamily, have both been found to be upregulated in patients with advanced stages of cancer and various chronic diseases such as kidney disease, congestive heart failure, diabetes, sarcopenia, and obesity [5,27]. They can bind to the activin type IIB receptor (ActRIIB), activating a cascade that leads to satellite cell quiescence and muscle degradation; see Figure 2A.

Myostatin has also been shown to activate MAPK/ERK in muscle precursor cells, which has the downstream effect of impairing the differentiation program [79]. The result is an accumulation of activated progenitor cells with impaired differentiation capabilities. Further, NF-κB has been identified as the target cachectic factors activate to deregulate Pax7 and inhibit differentiation [25]. Altogether, these findings suggest that cancer cachexia is the result of both increased muscle degradation and an impairment in satellite cell differentiation.

Pharmacological blockade of the ActRIIB pathway has been shown to partially reverse cancer-induced muscle wasting while having no effect on tumor growth or fat loss [31]. The soluble decoy receptor inhibits myostatin/activin A signaling, decreasing phosphorylated Smad2 which results in enhanced proliferation of satellite cells. It also increases the amount of phosphorylated FOXO3a and attenuates the expression of ubiquitin ligases responsible for muscle degradation; see Figure 2B. Zhou et al. [31] showed that this treatment can recover skeletal and heart muscle loss and increase survival times in several mouse models of cancer cachexia. Together with other work on myostatin [80], ActRIIB has been proposed as a promising new treatment approach for cachexia in cancer and other chronic diseases.

### 3.1. Muscle Loss in Cancer Cachexia

In our mathematical model, we considered three molecular signaling events that may contribute to muscle wasting in cancer cachexia that are downstream of myostatin and thus would be affected by ActRIIB blockade treatment. Cancer cells release systemic signaling factors that travel to muscle tissues through the circulation and activate the ActRIIB pathway. Through this and possibly other pathway activations, the following may occur:

1. satellite cells become quiescent or have a reduced proliferation rate [10,45],
2. satellite cells undergo apoptosis, necrosis, or may become permanently quiescent [81,82,83,84], and 
3. muscle cells atrophy [85].

Note that Item 2 above introduces the possibility of satellite cells dying. In our mathematical model, this addition allowed for the possibility of a satellite cell differentiation attempt that did not contribute to functional muscle fibers. Thus, it accounted for the possible impairment of the differentiation program with the loss of precursor cells, which has been shown to contribute to cancer cachexia [25,79].

We assumed the cachectic state was a perturbation away from the healthy state, tracing a disease evolution path through the parameter landscape. To incorporate the cachectic mechanisms into our mathematical model, we modified the healthy Equations (1)–(3) as follows. To begin, we modeled the growing tumor volume, *T*, with an exponential-linear model [86]. This choice reflected the fact that our experimental tumor growth data only demonstrated periods of exponential or linear growth [31]. Initially, there was a period of exponential growth with a growth rate μT. This was followed by a period of linear growth, with growth rate $\mu_{1}$:

$$\begin{array}{cccc} \frac{dT}{dt} & =\mu T(t), & T & ⩽T_{th}\\ \frac{dT}{dt} & =\mu_{1}, & T & >T_{th} \end{array}$$

The two growth periods are connected mathematically, as done in [86], by requiring $\mu T_{th}=\mu_{1}$, so that at the transition point $T_{th}$ the growth rates equal. For computational simplicity, the above two growth periods are combined into one ordinary differential equation, by introducing a transition parameter η:

(5) $$\frac{dT}{dt}=\mu T\left(t\right){\left(1+{\left(\frac{T(t)}{T_{th}}\right)}^{\eta }\right)}^{-\frac{1}{\eta }}=\mu T\left(t\right){\left(1+{\left(\frac{\mu }{\mu_{1}}T\left(t\right)\right)}^{\eta }\right)}^{-\frac{1}{\eta }}.$$

Here, η controls the speed at which the model transitions from the exponential phase to the linear phase of growth. As done in [86], we used η=20, which was sufficiently fast to capture the transition observed in the data.

Next, we incorporated tumor-induced satellite cell quiescence and interpreted this as a suppression of the proliferation rate ν(t). Thus, cancer reduction of proliferation, which already depends on muscle mass size, was modified by the multiplicative factor $\left(1-\varepsilon \frac{T(t)}{m_{2}+T\left(t\right)}\right)$. This form allows the strength of the suppression to depend on tumor volume and can completely suppress proliferation regardless of the muscle feedback. It also ensures that the modified proliferation rate maintains positivity. In this suppression factor, ε is the maximum reduction possible (with 0≤ε≤1), and $m_{2}$ is a half-saturation constant for the tumor size dependent effect.

Finally, we incorporated tumor-induced satellite and muscle cell death by adding terms of the form $-d_{S}\frac{T(t)}{m_{2}+T\left(t\right)}S\left(t\right)$ to the satellite cell growth equation and $-d_{M}\frac{T(t)}{m_{2}+T\left(t\right)}M\left(t\right)$ to the muscle cell growth equation. Here, $d_{S}$ and $d_{M}$ are the tumor-induced satellite cell and muscle cell death rates, respectively, and $m_{2}$ is again a half-saturation constant. Modifying our healthy tissue model, Equations (1)–(3), gives the following mathematical description of the molecular mechanisms involved in cancer cachexia-induced muscle wasting:

(6) $$\begin{array}{cc} \frac{dS}{dt} & =\left(2p(t)-1\right)\nu \left(t\right)\left(1-\varepsilon \frac{T(t)}{m_{2}+T\left(t\right)}\right)S\left(t\right)-d_{S}\frac{T(t)}{m_{2}+T\left(t\right)}S\left(t\right), \end{array}$$

(7) $$\begin{array}{cc} \frac{dM}{dt} & =2\left(1-p(t)\right)\nu \left(t\right)\left(1-\varepsilon \frac{T(t)}{m_{2}+T\left(t\right)}\right)S\left(t\right)-\left(d_{0}+d_{M}\frac{T(t)}{m_{2}+T\left(t\right)}\right)M\left(t\right),\\ \mathrm{with} & \;p\left(t\right)=p_{0}+\frac{p_{1}}{1+\frac{1}{m}M\left(t\right)}\;\mathrm{and}\;\nu \left(t\right)=\nu_{0}+\frac{\nu_{1}}{1+\frac{1}{m}M\left(t\right)}. \end{array}$$

The above modifications were chosen in order to describe the cachectic state as a perturbation away from the healthy state. That is, the effect of the disease is to slowly push the system away from health (and the healthy parameter values) into a diseased state (and corresponding diseased parameter values). To do this, our model formulations involved functional forms that increased in efficacy as the tumor grew large. These same formulations in effect modified most of the healthy parameters by a scaling factor in the limit of a large tumor. For example, the natural death rate of muscle cells is $d_{0}$ in health, but $d_{0}+d_{M}$ when the tumor is very large. This choice to model disease as a perturbation from the healthy state allowed the “difference” between health and cachexia to be represented by the value of $d_{M}$, for example. In contrast, refitting all model parameters for the cachectic groups would require the difference d0cachexiay−d0healthy to represent the shift that occurs in the diseased state. An advantage of this perturbation approach is that the transition path from health to disease can be tracked through parameter space, which would not be possible when refitting parameters. Note that we assumed that the probability of stem cell self-renewal, p(t), is unchanged by cancer, but that cancer can alter the frequency of stem cell division, ν(t).

#### Parameterizing the Cachexia Model

**Figure 3:**
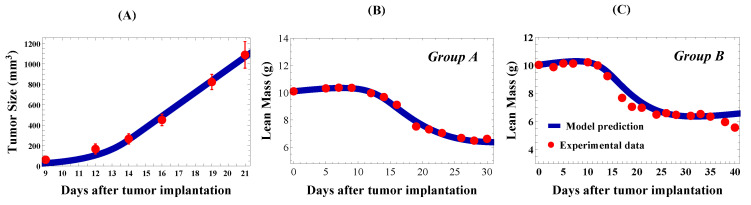
Tumor growth and cachexia model fits to experimental data [31]. (**A**) Exponential-linear tumor growth model (Equation (5), blue curve) fit to C26 experimental tumor volume data from [31] (red points). The initial condition for the numerical simulation was chosen to match the experimental conditions, with T(0)=0.5 mm${}^{3}$ (or $0.5\times 10^{6}$ cells) representing the initial tumor implant on Day 0. Model parameter values are listed in Table 2 (Row 1). (**B**) Cachexia model prediction (Equations (5)–(7), blue curve) fit to lean mass experimental data from Control Group A (red points). In (**C**), the model prediction was validated by comparing to lean mass experimental data from Control Group B (red points). Cachexia model parameter values are listed in Table 2 (Row 2), with initial conditions in Row 3 (Group A) and Row 4 (Group B). Numerical computations performed in Mathematica.

The cachexia mouse model on which we based our parameters is the C26 murine carcinoma model used in [31]. This is one of two commonly used animal models of cancer-induced cachexia [11]. The experimental setup involved subcutaneously injecting $0.5\times 10^{6}$ C26 cells into the rear flank of 10-week-old male CDF1 mice. We assigned the initial tumor volume to be T(0)=0.5 mm${}^{3}$ to match the implantation. To determine the exponential-linear growth rate parameters μ and $\mu_{1}$, we minimized the RMSE and fit the numerical solution of (5) to the experimental tumor volume measurements of [31]. The minimum RMSE was found by grid search over generous parameter ranges. The result of this fitting is shown in Figure 3A, with μ=0.446 days${}^{-1}$ and $\mu_{1}=116.0$ mm${}^{3}$ days${}^{-1}$. These rates gave a transition threshold of $T_{th}=260$ mm${}^{3}$, putting the transition from exponential to linear growth around Day 14. Exponential-linear tumor growth parameters are listed in Table 2 (Row 1).

In [31], two experimental groups were presented, one where anti-cachexia treatment started in “early cachexia” on Day 5 (Group A) and another where treatment started in “late cachexia” on Day 14 (Group B). To parameterize the cachectic state, we used the control from Group A and validated with the control from Group B. To determine the cachexia-inducing model parameters, ε, $d_{S}$, $d_{M}$, and $m_{2}$, we fit model predicted lean mass, ξ(M+S), from Equations (5)–(7) to Group A control data [31] by grid search to minimize the RMSE; see Figure 3B. The resulting cachexia parameter values are listed in Table 2 (Row 2). As done with the healthy body weight data, we decomposed the cachexia body weight measurements by assuming lean mass corresponded to 40% of the body mass after subtracting predicted tumor mass (based on Equation (5)) and the conversion factor ξ=0.002 g/mm${}^{3}$.

The system of Equations (5)–(7) was solved with initial conditions T(0)=0.5 mm${}^{3}$, S(0)=252.75 mm${}^{3}$, and M(0)=4799.25 mm${}^{3}$, and with the base model parameters from Table 1. The stem and muscle initial conditions correspond to the extrapolated M(t) and S(t) values from the body weight on injection day, using the predicted stem ratio from the healthy model, Equations (1)–(3), solved on Day 49, representing a 10-week-old male CDF1 mouse ($\psi_{49}=\frac{S(49)}{M(49)}=0.05266544$). For example, the average body weight of the 10 mice in Experimental Group A was about 25.26 g. Thus, we started the numerical simulation with:

(8) $$M\left(0\right)=\frac{0.4(25.26)}{(1+\psi_{49})\xi }\approx 4799.25\;{\mathrm{mm}}^{3}\;\mathrm{and}\;S\left(0\right)=\psi_{49}M\left(0\right)\approx 252.75\;{\mathrm{mm}}^{3}.$$

This ensured that the model prediction was fit to experimental data, while keeping the developmental stem ratio, and resulting feedback mechanisms, age-appropriate.

Fitting the model to Group A control data from [31] resulted in parameter values ε=0.004, $m_{2}=338$ mm${}^{3}$, $d_{S}=0.030$ day${}^{-1}$, and $d_{M}=0.104$ days${}^{-1}$ and the fit shown in Figure 3B. Comparison of our model prediction to Group B control data, with the same experimental setup, is shown in Figure 3C. All parameter values were the same between the two figures, with the exception of initial conditions for *M* and *S*. These were set according to (8) with the group’s average lean mass on Day 0, 25.10 g for Group B. Good agreement was observed between the model prediction and experimental control data in both groups.

The model fitting suggest that cachexia requires increased death rates to both muscle and satellite cells. The mechanism of satellite cell quiescence, however, is not required by the model to match experimental observations, as setting ε=0 results in approximately the same fit with the same RMSE; see Section 4.4. Alternatively, since the mass of the satellite compartment is quite small, the mechanism of quiescence may be appearing in our model as a death rate (permanent quiescence) rather than as a decrease in proliferation frequency. It is also possible that this loss from the stem compartment, without a corresponding gain to the muscle compartment, represents impaired differentiation, as found experimentally in [25,79].

### 3.2. Reversing Muscle Loss through Anti-Cachexia Treatment

Treatment with the soluble activin type IIB-receptor (sActRIIB) inhibits myostatin/activin A signaling and leads to dramatic muscle growth in vivo [31]. From Figure 2, we see that two downstream effects of blocking the ActRIIB pathway are satellite cell proliferation and enhanced muscle protein synthesis. Note that while the treatment partially reversed muscle wasting, it did not affect tumor growth in the C26 model.

To incorporate sActRIIB treatment into our mathematical model of cancer cachexia, we introduced treatment factors $A_{i}$ that limit the influence of the cachectic signals derived from the cancer. That is, Equations (5)–(7) were modified by introducing treatment parameters $A_{1}$, $A_{2}$, and $A_{3}$, which reduce the tumor-induced effects of ε, $d_{S}$, and $d_{M}$, respectively. Additionally, we introduced treatment parameter $A_{4}$, which allows the treatment to reduce the natural muscle death rate $d_{0}$. For each treatment parameter, we require $0\leq A_{i}\leq 1$, for i=1…4. The cachexia-treatment model is thus Equation (5) (unmodified by sActRIIB treatment) and:

(9) $$\begin{array}{cc} \frac{dS}{dt} & =\left(2p(t)-1\right)\nu \left(t\right)\left(1-A_{1}\varepsilon \frac{T(t)}{m_{2}+T\left(t\right)}\right)S\left(t\right)-A_{2}d_{S}\frac{T(t)}{m_{2}+T\left(t\right)}S\left(t\right), \end{array}$$

(10) $$\begin{array}{cc} \frac{dM}{dt} & =2\left(1-p(t)\right)\nu \left(t\right)\left(1-A_{1}\varepsilon \frac{T(t)}{m_{2}+T\left(t\right)}\right)S\left(t\right)-\left(A_{4}d_{0}+A_{3}d_{M}\frac{T(t)}{m_{2}+T\left(t\right)}\right)M\left(t\right). \end{array}$$

#### Parameterizing the Anti-Cachexia Treatment Model

Treatment parameters $A_{i}$ were determined by fitting this model to C26 + sActRIIB mouse data [31]. Two experimental groups were used: Group A where treatment started in early cachexia on Day 5 and Group B where treatment started in late cachexia on Day 14. To determine the model parameters, we again minimized the RMSE using a grid search and found the values reported in Table 2 Rows 5 and 6 for fits to Groups A and B, respectively.

**Figure 4:**
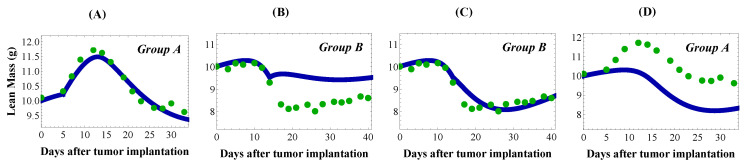
Model prediction of cachexia treatment in either early cachexia (Group A, treatment starting on Day 5) or late cachexia (Group B, treatment starting on Day 14). (**A**) The result of parameter fitting to Group A. (**B**) The prediction of Group B using Group A parameter values. (**C**) The result of parameter fitting to Group B. (**D**) The prediction of Group A using Group B parameter values. Experimental data (green dots) and model simulation (blue line). Numerical computations performed in Mathematica.

In both group fits, $A_{1}=A_{2}=0$, suggesting that treatment completely blocked the cancer-imposed stem death rate $d_{S}$ and the decrease in stem proliferation rate ε. Since ε≈0 from our cachexia fit, the treatment fit of $A_{1}=0$ was not surprising. The finding that $A_{2}=0$ suggested that treatment reactivated the quiescent satellite cell population and/or repaired the dysfunctional differentiation program. In Group A’s fit, we found $A_{3}=0.51$ and $A_{4}=0.54$, corresponding to a decrease in both the cancer-imposed muscle death rate $d_{M}$ and the natural muscle death rate $d_{0}$. Decreasing the natural death rate allowed the model to predict the bump in lean mass observed around Day 13 in Group A; see Figure 4A. However, fitting to Group B, which did not present such an obvious bump (Figure 4C), we found $A_{3}=0.62$ and $A_{4}=1$, corresponding to a decrease in the cancer-imposed death rate $d_{M}$ and no treatment alteration of the natural death rate $d_{0}$.

## 4. Results

We now present the results of the model analyses and the implications for satellite cell and muscle cell behaviors in healthy, cachectic, and anti-cachectic treatment conditions.

### 4.1. The Healthy Muscle Stem Cell Ratio

During the adolescent growth stage, satellite cells proliferate to increase muscle mass. As the muscle matures, satellite cells become inactive and are held in reserve until needed for muscle repair. Skeletal muscle in adulthood is generally stable with the numbers of satellite and muscle cells relatively unchanged. Under these normal adult conditions, our mathematical model gives the following steady-state:

(11) $$\begin{array}{cccc} S_{ss} & =\frac{2md_{0}p_{1}\left(2(p_{0}+p_{1})-1\right)}{\left(1-2p_{0}\right)\left(2p_{1}\nu_{0}+\left(1-2p_{0}\right)\nu_{1}\right)}, & M_{ss} & =\frac{m\left(2(p_{0}+p_{1})-1\right)}{1-2p_{0}}. \end{array}$$

**Figure 5:**
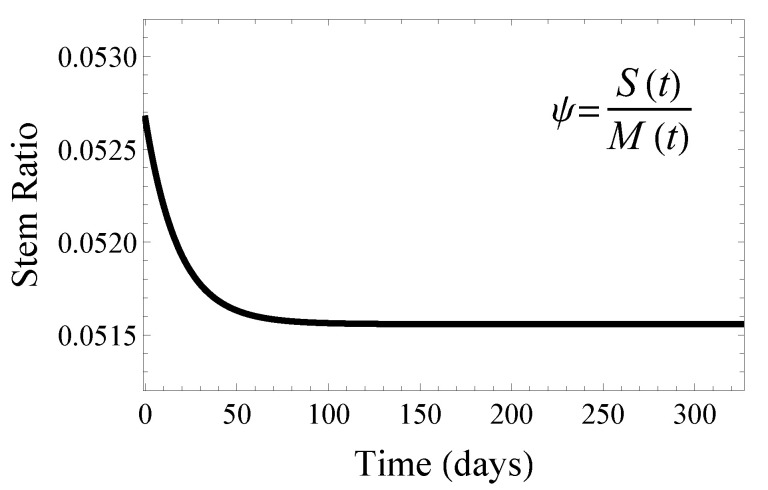
Model simulation of the stem cell ratio $\psi \left(t\right)=\frac{S(t)}{M(t)}$. The ratio started higher due to our estimated initial conditions and the period of rapid growth in the first 3–15 weeks of life. Once the adult lean mass was obtained, the stem ratio reached the steady-state homeostatic value. Numerical computations performed in Mathematica.

The stem cell ratio in the steady-state is thus

(12) $$\psi_{ss}=\frac{S_{ss}}{M_{ss}}=\frac{2d_{0}p_{1}}{2p_{1}\nu_{0}+\left(1-2p_{0}\right)\nu_{1}},$$

which has a natural upper bound of $\frac{d_{0}}{\nu_{0}}$, the ratio of the natural muscle cell death rate to the satellite cell homeostatic proliferation rate. Using our fitted parameter values in Table 1, the steady-state stem ratio is about $\psi_{ss}=0.0516$. See Figure 5 for the model predicted stem cell ratio for muscle tissue in mice three weeks and older.

Sensitivity analysis of the healthy model was performed by computing the relative sensitivity of satellite and muscle compartments to small changes in input parameter values. The homeostatic steady-state, (11), was used as the base condition. We then computed the relative change in $S_{ss}$ and $M_{ss}$ given a 5% increase or decrease in the parameter values according to the formula:

(13) $$\begin{array}{c} \phi =\frac{y\left(\rho (1\pm 0.05)\right)-y\left(\rho \right)}{y(\rho )}100\%, \end{array}$$

where *y* is either $S_{ss}$ or $M_{ss}$ and ρ is any model parameter from Table 1. The sensitivity results are summarized in Table 3. The most sensitive model parameter is the homeostatic probability of self-renewing division $p_{0}$. As discussed above, when $p\left(t\right)>\frac{1}{2}$, the stem compartment grows without bound, so this result is to be expected.

### 4.2. Wound Healing in the Healthy State

After damage, even for severe and repeated injuries, skeletal muscle has a remarkable regenerative capacity. Pro-inflammatory signals from the wound area activate satellite cells to proliferate, providing myoblasts to repair muscle fibers.

**Figure 6:**
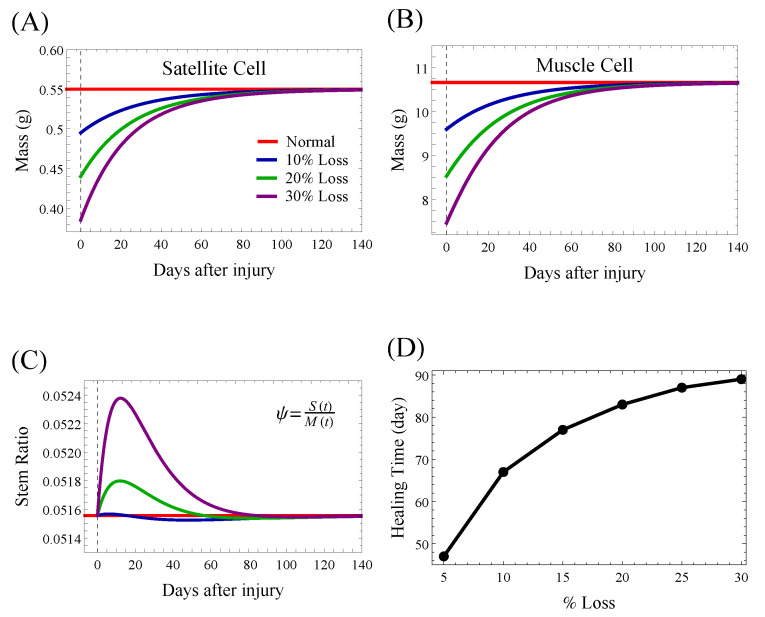
Model prediction of the skeletal muscle response to an injury of 10, 20, or 30% loss in each model compartment; that is, the initial conditions were set to $S\left(0\right)=\chi S_{ss}$ and $M\left(0\right)=\chi M_{ss}$, where χ=0.9 for a 10% loss, 0.8 for a 20% loss, and 0.7 for a 30% loss of mass. Pre-injury, the muscle cells are at the homeostatic level. Post-injury, the satellite cells are dividing to renew themselves (**A**) and differentiate into new muscle cells (**B**), and both compartments slowly return to their homeostatic (pre-injury) levels. (**C**) The stem ratio increases transiently in response to the injury. (**D**) Healing time for skeletal muscle after an injury to both muscle and satellite model compartments. Healing time is defined by the time required to re-obtain the pre-injury homeostatic mass computationally as $\left|M\left(t\right)-M_{SS}\right|<0.1$. Numerical computations performed in Mathematica.

To test the wound healing ability of our model, we considered three injuries of increasing severity: a loss of 10, 20, or 30% in both satellite and muscle cell compartments. Figure 6A shows the satellite cell dynamic, which increases to regenerate the muscle and then slowly returns to its homeostatic (pre-injury) level. As a result of satellite cell proliferation, the number of muscle cells increases until reaching the homeostatic level; see Figure 6B. The number of satellite cells depends on the severity of the lesion; see Figure 6C. The time required to heal the wound depends on the severity of the injury, as shown in Figure 6D. Larger injuries require longer healing times, according to a sub-linear relationship. The more serious the injury, the longer it takes to re-achieve homeostasis.

### 4.3. Sensitivity Analysis for Cachexia and Treatment

Sensitivity analyses for the cachexia (5)–(7) and treatment models (5), (9) and (10) are now presented. The relative change in muscle or satellite cell compartments is reported on Day 20 post-implant to determine the sensitivity during the dynamic period of muscle loss (rather than the steady-state, which is likely not obtained by the cachectic animal). Similar to the above, we iteratively considered a 5% increase or decrease to the cachexia or treatment base parameter values, using (13) to compute the relative change.

**Figure 7:**
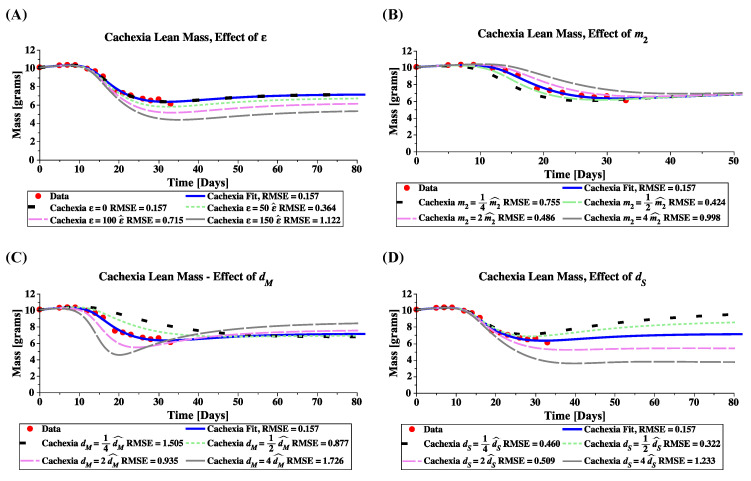
Model simulations of lean mass showing the effect of cachexia parameters ε (**A**), $m_{2}$ (**B**), $d_{M}$ (**C**), and $d_{S}$ (**D**). Parameter value estimates $\hat{\varepsilon }$, $\hat{m_{2}}$, $\hat{d_{M}}$, and $\hat{d_{S}}$ are as listed in Table 2 (Row 2), unless otherwise specified. All simulations used initial conditions for Group A. Numerical computations performed in Maple.

Under cachexia, the top of Table 4, the model is most sensitive to the tumor-induced death rates for satellite cells ($d_{S}$) and muscle cells ($d_{M}$). The satellite cell population is most sensitive to $d_{S}$, as this parameter directly affects S(t), while the death rate for muscle cells affects the stem population indirectly through the feedback mechanisms. The muscle mass is most sensitive to parameter $d_{M}$, with $m_{2}$ the second most sensitive, as this parameter shifts the half-saturation point of the tumor’s influence. Note that since parameter ε (tumor-induced quiescence) was very close to zero, its sensitivity was also essentially zero. If the base value of ε were larger, we may see a more significant sensitivity here (see Figure 7).

Under treatment, bottom of Table 4, we left out parameters $A_{1}$ and $A_{2}$ since they were estimated to have values of zero. We report only sensitivity for parameter $A_{3}$, the treatment reduction of tumor-induced muscle death rate, and parameter $A_{4}$, the treatment reduction of the natural death rate. We used parameter estimates obtained from fitting to Experimental Group A, when treatment was given early in cachexia on Day 5. Again, sensitivity was performed on Day 20 post-implant to capture the dynamical region of muscle loss and recovery. Both compartments are more sensitive to $A_{4}$ (reduction to the natural death rate) than to parameter $A_{3}$ (reduction to the tumor-induced death rate), but this difference is likely not significant as their mechanisms of action in the model are similar.

### 4.4. Mechanisms of Cachexia Target Muscle or Satellite Cells With Different Effects

Simulations of the cachexia model, Equations (5)–(7), demonstrate that the various mechanisms of cachexia (represented by model parameters ε, $m_{2}$, $d_{M}$, and $d_{S}$) have differing biological implications, as shown in Figure 7. Briefly, targeting muscle cells directly through an increased death rate leads to rapid mass loss which is recovered in time; whereas targeting satellite cells by inducing quiescence or death leads to a permanent loss of lean mass.

**Figure 8:**
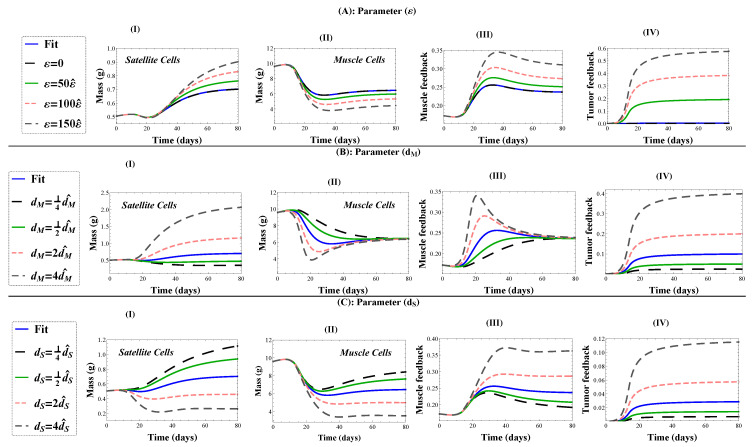
Model simulations showing the effect of cachexia parameters ε (**Row A**), $d_{M}$ (**Row B**), and $d_{S}$ (**Row C**) on satellite cells (**Column I**), muscle cells (**Column II**), the feedback function in Equations (2) and (3) (**Column III**), and the tumor-induced muscle death function (**Column IV**). Parameter value estimates $\hat{\varepsilon }$, $\hat{d_{M}}$, and $\hat{d_{S}}$ are as listed in Table 2 (Row 2), unless otherwise specified. Muscle feedback is the function $\frac{1}{1+\frac{1}{m}M\left(t\right)}$, and tumor feedback is the function $\frac{T(t)}{m_{2}+T\left(t\right)}$ multiplied by ε in (**AIV**), $d_{M}$ in (**BIV**), and $d_{S}$ in (**CIV**). All simulations used initial conditions for Group A. Numerical computations performed in Mathematica.

Increasing parameter ε in the satellite cell proliferation suppression factor $\left(1-\varepsilon \frac{T(t)}{m_{2}+T\left(t\right)}\right)$ increases the total loss of lean mass; see Figure 7A. Note that our estimated value of ε gives the same prediction as ε = 0, suggesting that this mechanism is not being used to induce cachexia. According to the assumed negative feedback mechanism that directly affects the proliferation probability and division rate of satellite cells through the function $\left(\frac{1}{1+\frac{1}{m}M\left(t\right)}\right)$ in Equations (2) and (3), the muscle cell reduction triggers the release of signals that stimulate satellite cell proliferation and differentiation; see Figure 8(AI,AII). The greater the amount of muscle loss in the body, the stronger the activated feedback signaling; see Figure 8(AIII). The feedback proliferation is simultaneously suppressed by the tumor-host interaction factor $\left(1-\varepsilon \frac{T(t)}{m_{2}+T\left(t\right)}\right)$, which scales the division rate ν(t) in Equations (6) and (7), and turns satellite cells quiescent. As  ε increases, the inhibitory effect on satellite cell proliferation is larger; see Figure 8(AIV). Therefore, the satellite compartment is not able to maintain the healthy steady-state, and as a result, mass is lost.

Increasing the half saturation constant $m_{2}$ delays the transition from health to cachexia (Figure 7B), as larger tumors are required to induce the same level of signaling. Parameter $m_{2}$ does not, however, affect the equilibrium value of the cachectic state.

Increasing parameter $d_{M}$, the tumor-induced muscle death rate, leads to a rapid loss of muscle mass in the body; see Figure 8(BII). As a result, satellite cells are activated by the feedback mechanism, to self-renew and create new healthy muscle fibers; see Figure 8(BI). The larger the tumor-induced death rate, the larger the feedback effect; see Figure 8(BIII). This response by the satellite cells partially recovers the muscle mass loss, but requires a significant increase in satellite mass, resulting in a higher steady-state mass, which is likely not realized by the host; see Figure 7C. Importantly, the model predicts that while an increased muscle death rate could lead to rapid mass loss, the effect is not lasting, and mass would be recovered by the stem cells given sufficient time.

Increasing parameter $d_{S}$, the tumor-induced satellite cell death rate, decreases the stem cell reserve (Figure 8(CI)) and results in a reduced muscle cell mass at steady-state; see Figure 8(CII). As muscle mass drops, the feedback system attempts to activate satellite cells; see Figure 8(CIII). The tumor-induced stem death rate is a significant mechanism of lean mass loss that can not be overcome by feedback activation; see Figure 8(CIV). As a result, lean mass steady-state decreases with increasing tumor-induced stem death rate; see Figure 7D.

### 4.5. Treatment Partially Restores Muscle Mass by Reactivating Satellite Cells

**Figure 9:**
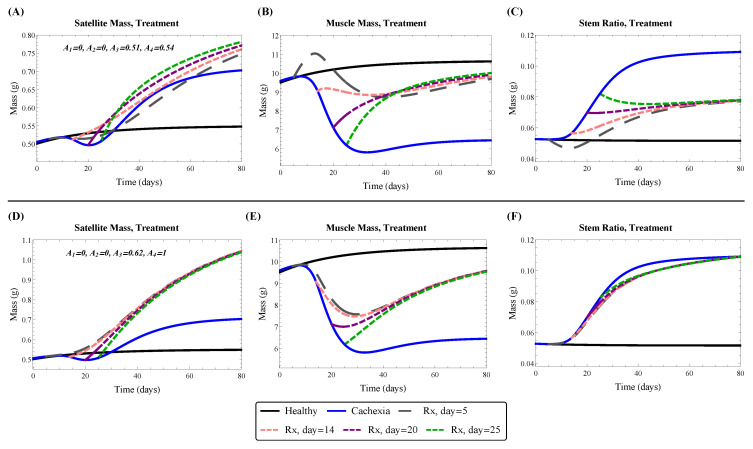
Model predicted satellite cell mass (**A**,**D**), muscle mass (**B**,**E**), and stem ratio (**C**,**F**) under cachexia treatment starting on Day 5, 14, 20, or 25 after tumor implantation. The top row (**A**–**C**) uses treatment parameters from fitting to Group A. The bottom row (**D**–**F**) uses treatment parameters from fitting to Group B. Numerical computations performed in Mathematica.

Using our model of cachexia treatment, we now examine the effects of treatment on lean mass. Figure 9 shows the model prediction using treatment parameter values determined by fitting with either Group A, where mice were treated in early cachexia on Day 5, or Group B, where mice were treated in late cachexia on Day 14. The parameter values are as listed in Table 2. In both fits, the treatment blocked cachexia mechanisms targeting satellite cells, resulting in their reactivation; see Figure 9A,D. The mechanisms targeting muscle cells were reduced by about 50% in efficacy. The main difference between the fits was that the natural muscle death rate was reduced in Group A (in order to capture the lean mass bump observed post-treatment), whereas it was not affected at all in Group B; see Figure 9B,E.

Removal of the inhibition of satellite cells by cachexia treatment resulted in their reactivation and proliferation to replenish the lost muscle mass. With treatment only partially blocking muscle cell death, the natural feedback pushed the system to obtain a new steady-state where lean mass was below the healthy level and the stem ratio was higher; see Figure 9C,F. Interestingly, when the natural muscle death rate was reduced by treatment, Group A fit, the stem ratio converged to about 8%, while it converged to the predicted cachexia stem ratio of 10% when the natural death rate was unaffected by treatment, Group B fit. This is a result of the maximal muscle death rates (natural plus cachexia), which were:

$$\begin{array}{cc} \; & \mathrm{Group}\;A\;\mathrm{fit}\text{:}\;A_{4}d_{0}+A_{3}d_{M}=0.54\left(0.05\right)+0.51\left(0.104\right)=0.08\\ \; & \mathrm{Group}\;B\;\mathrm{fit}\text{:}\;A_{4}d_{0}+A_{3}d_{M}=1\left(0.05\right)+0.62\left(0.104\right)=0.11. \end{array}$$

Thus, the model predicts a higher muscle death rate when treatment occurs in late cachexia than when treatment occurs earlier. Both of these rates are still higher than the natural death rate, which results in a lean mass steady-state smaller than in the healthy state.

## 5. Discussion

In this study, we mathematically investigate cancer-associated muscle wasting through the intracellular ActRIIB signaling pathway. Using a stem-hierarchy framework, we presented a new model of cancer cachexia and treatment that was parameterized with C26 carcinoma experimental data. We extended our cancer cachexia model to examine the consequences of anti-ActRIIB treatment causing a partial reversal of muscle loss due to satellite cell activation and limited muscle degradation. In cancer cachexia, signaling pathways other than the myostatin/activin A pathway have been investigated [24,25,87,88]. However, the inhibition of the myostatin/activin A pathway can also reverse muscle loss in other diseases such as Duchenne muscular dystrophy [89,90,91,92], metabolic diseases (obesity and diabetes) [93,94], androgen deficiency [95], X-linked myotubular myopathy [96], amyotrophic lateral sclerosis [97], and limb-girdle muscular dystrophy [98].

The mathematical model was parameterized in three stages: health, cachexia, and anti-cachexia treatment. The feedback mechanisms of the healthy state were determined by simulated annealing to minimize the RMSE. The disease and treatment parameters were determined by grid search over generous parameter ranges to again minimize the RMSE. The choice of the RMSE as the optimization function is standard, but did affect our parameterization results, and thus simulation results. Further, while all attempts to find the global minima in the simulated annealing algorithm were taken, it is a stochastic algorithm and thus may have found a local minimum instead. The values of the parameters we used in this study would affect our sensitivity results in Table 3 and Table 4. For all parameters except $p_{0}$, we believe the sensitivity was satisfactory. The high sensitivity to changes in $p_{0}$ was due to our choice to exclude asymmetric stem cell division in favor of reducing the complexity of our model. This is an area of improvement that may be considered in future work.

Our model predictions were consistent with previously published and experimentally validated work. In [99], the authors proposed that myostatin and activins are capable of binding to both ActRIIA and ActRIIB (activin type II receptors) with different affinities and that to achieve a strong functional benefit, the blockade of both receptors is needed. The experimental data used here assumed only the effect of ActRIIB pathway blockade, and thus, so did our modeling work. Future work can extend the model here to consider ActRIIA pathway blockade, which may lead to greater muscle regrowth and full recovery of tumor-induced loss. Furthermore, images of muscle fiber cross-sections [31] suggest that muscle atrophy also involves shrinkage of fiber diameters, which is another mechanism of mass loss that was not considered in our cachexia model. This is potentially an important mechanism of mass loss that should be included in the model and is the subject of future work. We speculate that inclusion of this alternate mechanism may help to explain the bump observed in lean mass recovery after early treatment of cachexia, which was not present in late treatment.

The modeling work presented here suggested that anti-cachexia treatment could partially recover muscle mass loss by reactivating satellite cells, resulting in a higher than normal stem ratio. The fact that satellite cell reactivation and differentiation were significant to muscle recovery under treatment was consistent with the experimental evidence reported in [31]. However, other studies have reported that muscle hypertrophy after pharmacological inhibition of the myostatin pathway occurs without significant activation of satellite cells and that therefore the satellite cells play little to no role in muscle hypertrophy when induced by this pathway [100,101,102,103]. Since the satellite cells are primitive cells that regulate muscle homeostasis, this suggests that myostatin and activin A may predominantly signal directly to myofibers. Here, we focused on the stem cell hierarchy. The distinctive proliferative behaviors of stem cells emerged as a result of the feedback dynamics, and hence, muscle regrowth was a direct consequence of stem cell regenerative potential. If these signaling molecules directly alter myofibers, possibly in altering fiber diameter, then as discussed above, this could be explored in an extension of this work. Future work can also explore the effects of other factors or agents that potentially act as intermediates in signal transmission within the stem cell hierarchy.

Our study examined cancer-induced muscle loss by considering the actions of cachectic factors on muscle satellite cells and fully differentiated muscle fibers. The significance of muscle precursor cells and the cancer-induced impairment of the differentiation program, as modulated by Pax7, comprise an area of interest and are left to future work. Furthermore, our use of ordinary differential equations enabled the thorough analysis we presented, but limited the biology that we could capture. For example, tissue architecture may play a crucial role in a muscle’s response to cachexia. Cachectic factors produced by cancer cells travel to muscles via blood. Thus, the amount of muscle vascularization and the proximity of satellite cells to blood vessels may help differentiate how skeletal muscle responds versus heart muscle, for example. To explore the role these spatial biological features may play in cachexia, a partial differential equation-based model may be appropriate.

Indeed, this model was already difficult to parameterize due to the limited types of data (i.e., whole body weight, but no breakdown into satellite cell behavior). Future work can expand on this model by incorporating new biological mechanisms that require more complex mathematical techniques as the required data become available.

A deeper understanding of the mechanisms underlying cancer cachexia is needed to evaluate the efficacy of potential therapies including novel immunomodulatory or metabolic targets. Aside from being the direct effect of tumor growth, cancer cachexia is associated with a collection of systemic dysfunctions including inflammation [19,20,104] and metabolic alterations such as increased insulin resistance, increased energy expenditure, and increased heat generation via defective mitochondrial metabolism and fat tissue browning [105,106]. Therefore, advancing our cachectic model to include inflammation, metabolic alterations, and/or adipose tissue may provide new insights into the significant mechanisms and systemic cross-talk underlying cancer cachexia.

## Figures and Tables

**Table 1:** Parameter values for the healthy muscle model Equations (1)–(3) found by fitting the model prediction to the estimated and extended muscle and satellite mass data of the CDF1 male mouse. Simulated annealing was used to find $p_{0}$, $p_{1}$, $\nu_{0}$, and $\nu_{1}$, while *d* and *m* were iterated over manually. The parameters listed correspond to the best fit (minimum RMSE = 0.168). Initial conditions were estimated for a 3-week-old CDF1 male mouse.

Model Parameters		
$p_{0}=0.479$	$v_{0}=0.087$ day${}^{-1}$	$d_{0}=0.05$ day${}^{-1}$
$p_{1}=0.133$	$v_{1}=5.591$ day${}^{-1}$	m=1000 mm${}^{3}$
Initial Conditions		
S(0)=0.536 g	M(0)=10.17784 g	ξ=0.002 g/mm${}^{3}$
S(0)=268.01 mm${}^{3}$	M(0)=5088.92 mm${}^{3}$	

**Table 2:** Model parameter values and initial conditions. Row 1: Parameter values for the exponential-linear model of tumor growth, (5), RMSE = 34.11. Row 2: Parameter values for the cachexia model, (6) and (7), RMSE = 0.157. Rows 3–4: Initial conditions for Experimental Groups A and B, respectively, according to (8). Row 5: Parameter values for cachexia treatment by fitting to Experimental Group A, (9) and (10), RMSE = 0.176. Row 6: Parameter values for cachexia treatment by fitting to Group B, RMSE = 0.317. Parameter values determined by curve fitting the model solution to experimental data from [31] for C26 tumor-bearing mice with or without sActRIIB treatment. Best fit determined by minimizing the RMSE between predicted muscle and satellite mass and experimental lean mass data.

	Model Equations	Fitted Parameter Values			
1.	Exp-linear model (5)	μ=0.446 days${}^{-1}$	$\mu_{1}=116.0$ mm${}^{3}$days${}^{-1}$	η=20	T(0)=0.5 mm${}^{3}$
2.	Cancer-Cachexia (6) and (7)	ε=0.004	$d_{S}=0.030$ days${}^{-1}$	$d_{M}=0.104$ days${}^{-1}$	$m_{2}=338$ mm${}^{3}$
3.	Group A ICs	$M^{A}\left(0\right)=4799.25$ mm${}^{3}$		$S^{A}\left(0\right)=252.75$ mm${}^{3}$	
4.	Group B ICs	$M^{B}\left(0\right)=4768.85$ mm${}^{3}$		$S^{B}\left(0\right)=251.15$ mm${}^{3}$	
5.	sActRIIB Treatment (9) and (10) Group A	$A_{1}=0$	$A_{2}=0$	$A_{3}=0.51$	$A_{4}=0.54$
6.	Group B	$A_{1}=0$	$A_{2}=0$	$A_{3}=0.62$	$A_{4}=1$

**Table 3:** Sensitivity analysis for healthy muscle parameters. The relative change in satellite or muscle compartments is reported given a 5% increase or decrease of the base model parameter value from Table 1. Sensitivity is performed in the steady-state condition, (11).

Parameter	Relative Change Satellite Cells S(t)		Relative Change Muscle Cells M(t)	
ρ	5% Decrease	5% Increase	5% Decrease	5% Increase
m=1000	−5%	5%	−5%	5%
$p_{0}=0.479$	−82.0%	22,541.6%	−63.3%	−964.1%
$p_{1}=0.133$	−10.2%	10.7%	−5.9%	5.9%
$\nu_{0}=0.087$	0.45%	−0.45%	NA	NA
$\nu_{1}=5.591$	4.8%	−4.4%	NA	NA
d=0.05	−5%	5%	NA	NA

**Table 4:** Sensitivity analysis for cachexia and treatment model parameters. The relative change in satellite or muscle compartments is reported given a 5% increase or decrease to the estimated value from Table 2 (Rows 2 and 5). Sensitivity was performed on Day 20 post-tumor implantation to capture sensitivity during the dynamical range of interest (during muscle loss).

Cachexia Parameters				
Parameter	Relative Change Satellite Cells S(20)		Relative Change Muscle Cells M(20)	
ρ	5% decrease	5% increase	5% decrease	5% increase
$m_{2}=338$	−0.074%	0.074%	−0.71%	0.69%
ε=0.004	0.00	0.00	0.00	0.00
$d_{S}=0.030$	0.72%	−0.72%	0.16%	−0.16%
$d_{M}=0.104$	−0.46%	0.46%	1.55%	−1.51%
Treatment Parameters (Fit to Group A)				
parameter	5% decrease	5% increase	5% decrease	5% increase
$A_{3}=0.51$	−0.15%	0.15%	0.95%	−0.94%
$A_{4}=0.54$	−0.35%	0.36%	1.11%	−1.09%

## Abbreviations

   The following abbreviations are used in this manuscript:

ActRIIB	activin type-2B receptor
C26	colon-26 adenocarcinoma mouse model
CDF1 mouse	a cross between female BALB/cAnNCrl and male DBA2NCrl mice
FOXO3a	forkhead box 03A
IL-1, IL-6, IL-10	interleukin-1, interleukin-6, interleukin-10
MAPK/ERK	mitogen-activated protein kinase/extracellular signal-regulated kinase
MuRF1	muscle RING-finger protein-1
NF-κB	nuclear factor κB
Pax7	paired box 7
RMSE	root-mean-squared-error
sActRIIB	soluble decoy Activin type-2B receptor
SMAD2	mothers against decapentaplegic homolog 2
STAT3	signal transducer and activator of transcription 3
TGF-β	transforming growth factor β
TNFα	tumor necrosis factor α

## Appendix A. Numerical Solution of Anti-Cachexia Treatment Model

Included here is a Mathematica (www.wolfram.com/mathematica/) script to perform the numerical simulation of the anti-cachexia treatment model as shown in Figure 4. The simulation uses parameter values from fitting with experimental treatment Group A with tumor injection on Day 0 and administration of sActRIIB treatment on Day 5.

*(*Define the healthy model parameters:*)*

p0 = 0.479; p1 = 0.133; nu0 = 0.087; nu1 = 5.591;

m = 1000; d0 = 0.05; phi = 0.002;

*(*Define the tumor growth model parameters:*)*

mu = 0.446; mu1 = 116.0; eta = 20;

*(*Define the cachexia model parameters:*)*

m2 = 338; epsilon = 0.004;

dS = 0.030; dM = 0.104;

*(*Define the model differential equations:*)*

Eqns = {T’[t] == mu * T[t] * (1 + (mu * T[t]/mu1)^eta )^(-1/eta),

 S’[t] == (2 * (p0 + p1/(1 + M[t]/m)) - 1) * (nu0 + nu1/(1 + M[t]/m)) *

   (1 - A1 * epsilon * T[t]/(m2 + T[t])) * S[t]

   - A2 * dS * T[t]/(m2 + T[t]) * S[t],

 M’[t] == 2 * (1 - p0 - p1/(1 + M[t]/m)) * (nu0 + nu1/(1 + M[t]/m)) *

   (1 - A1 * epsilon * T[t]/(m2 + T[t])) * S[t]

   - (A4 * d0 + A3 * dM * T[t]/(m2 + T[t])) * M[t] };

*(*Define the model variables:*)*

Var = {T, S, M};

*(*Define initial conditions for tumor injection on day 0:*)*

ICs1 = {T[0] == 0.5, S[0] == 252.75, M[0] == 4799.25};

*(*Set A1=A2=A3=A4=1 since no treatment on days 0-5:*)*

A1=1; A2=1; A3=1; A4=1;

*(*Solve the initial value problem for cachexia no treatment over days 0-5:*)*

sol1 = **NDSolve**[{Eqns, ICs1}, Var, {t, 0, 5}];

*(*Define initial conditions for treatment start on day 5:*)*

T5 = T[5] /. sol1[[1]]; S5 = S[5] /. sol1[[1]]; M5 = M[5] /. sol1[[1]];

ICs2 = {T[5] == T5, S[5] == S5, M[5] == M5};

*(*Evaluate solution with A1, A2, A3, A4 Group A treatment values:*)*

A1=0; A2=0; A3=0.51; A4=0.54;

*(*Solve the initial value problem for anti-cachexia treatment over days 5-33:*)*

sol2 = **NDSolve**[{Eqns, ICs2}, Var, {t, 5, 33}];

*(*plot model simulations converted to lean body mass in [gram]*)*

P1 = **Plot[Evaluate**[phi * (S[t] + M[t]) /. sol1 ],{t,0,5}, **PlotRange** -> {{0, 33}, {9, 12}},

 **PlotStyle** -> {**Blue**, **Thickness**[0.02]}];

P2 = **Plot[Evaluate**[phi * (S[t] + M[t]) /. sol2 ],{t,5,33},**PlotStyle** -> {**Blue**, **Thickness**[0.02]}];

**Show**[P1, P2, **Frame** -> **True**, FrameTicksStyle -> Directive[**Black**, 20],

 **FrameLabel** -> {Style[“Days after tumor implantation”, 22, Bold],

 Style[“Lean Mass (g)”, 22, Bold]},

 LabelStyle -> Directive[**Black**, 18, **FontFamily** -> “Times New Roman”], **ImageSize** -> 400,

 **Epilog** -> **Text**[Style[“Group A”, **Black**, Bold, Italic, 24,

 **FontFamily** -> “**Times** New Roman”], **Scaled**[{.8, .85}]]]
